# Budget impact analysis of the subcutaneous infliximab (CT-P13 SC) for treating inflammatory bowel disease in the Big-5 European (E5) countries

**DOI:** 10.1186/s12913-022-08683-y

**Published:** 2022-11-04

**Authors:** Hyun Kyeong Yoo, Han Geul Byun, Flavio Caprioli, Mathurin Fumery, Laurent Peyrin-Biroulet, Subramanian Sreedhar, James Potter, Minyoung Jang

**Affiliations:** 1 Celltrion Healthcare, 19, Academy-ro 51, Yeonsu-gu, Incheon, South Korea; 2 Department of Pathophysiology and Transplantation, Università Degli Studi Di Milano, Milan, Italy; 3 Gastroenterology and Endoscopy Unit, Fondazione IRCCS Cà Granda, Ospedale Maggiore Policlinico Di Milano, Milan, Italy; 4 Service Hépato-Gastroentérologie, CHU Amiens Picardie, Amiens, France; 5 Department of Gastroenterology, Nancy University Hospital, Nancy, France; 6 Department of Gastroenterology, Royal Liverpool and Broadgreen University Hospitals NHS Trust, Liverpool, UK; 7 University of Liverpool, Liverpool, UK; 8 Celltrion Healthcare, Slough, UK

**Keywords:** Inflammatory bowel disease, Ulcerative colitis, Crohn’s disease, Budget impact

## Abstract

**Background:**

In 2020, the European Medicines Agency approved infliximab subcutaneous (SC) for the treatment of inflammatory bowel disease. This new mode of infliximab administration will reduce outpatient visits and costs of intravenous (IV) administration. This article describes a budget impact analysis of introducing infliximab SC to the Big-5 European (E5) market (Germany, France, Italy, Spain and UK) for 5 years, from the healthcare payer’s perspective.

**Methods:**

A prevalence-based budget impact model was developed to examine the financial impact of infliximab SC. “World with” versus “world without” infliximab SC scenarios were compared, including the potential administration costs of IV administration.

**Results:**

Introducing infliximab SC in patients with Crohn’s disease (CD) for 5 years resulted in cost savings of €42.0 million in the UK, €59.4 million in Germany, and €46.4 million in France and Italy, but increased budget expenditure in Spain by €3.8 million. For ulcerative colitis (UC), cost savings of €42.7 million in the UK, €44.9 million in Germany, €44.3 million in France, and €53.0 million in Italy occurred, but with no savings in Spain for 5 years. Cost-savings per patient was calculated by diving the net budget saving by number of treatment eligible patients. Maximum and minimum saving per patient per year ranged between €38.25 and €575.74 in CD, both from Germany, and €105.06 (France) and €647.25 (Germany) in UC.

**Conclusion:**

Healthcare payers in the UK, Germany, France, and Italy, but not in Spain, will make budget savings by using infliximab SC for the treatment of inflammatory bowel disease.

**Supplementary Information:**

The online version contains supplementary material available at 10.1186/s12913-022-08683-y.

## Background

Characterized by chronic inflammation of the gastrointestinal (GI) tract, the two most common types of inflammatory bowel disease (IBD) are Crohn’s disease (CD) and ulcerative colitis (UC) [1]. IBD is an immune-mediated inflammatory disease with symptoms including diarrhoea, abdominal pain, fatigue, and unintended weight loss [2]. CD is characterized by a transmural inflammation pattern, while the inflammation of UC is restricted to the mucosa [3]. The chronic inflammation associated with IBD can cause extensive and irreversible tissue damage, as well as complications leading to intestinal resection and disability, thus greatly compromising patients’ quality of life. Additionally, both CD and UC can interfere with normal GI function, potentially leading to malnutrition and vitamin and mineral deficiencies [4]. Most people with IBD experience periods of high disease activity (defined as relapses or flare-ups), interspersed by periods of remission; however, 10–15% of people have chronic, continuous disease activity [5].

IBD affects around 2.2–3.0 million people in Europe [6, 7]. It has been estimated that up to 1.6 million people in Europe live with CD, and up to 2.1 million people have UC [7]. Reported prevalence rates in Europe are among the highest globally [8, 9]. In 2017, the overall estimated prevalence of IBD in Europe was 0.16%, with country-specific rates in the E5 countries varying from 0.07% in Spain to 0.54% in the UK [10]. Specifically, a large variation in prevalence rates (CD 1.5–213 cases, and UC 2.4–294 cases, per 100,000 people) has been observed in different European studies [7].

The conventional therapeutic approach to IBD is progressive intensification of treatment based on disease severity [11, 12]. The most commonly used drugs are 5-aminosalicylates, corticosteroids, immunosuppressives, biologics, anti-integrin, and Janus kinase inhibitors [13]. Biologics specifically target key immunological and inflammatory pathways, allowing selective but highly potent control of inflammation [14]. Biological treatments currently approved for IBD treatment in Europe include anti-tumour necrosis factor (TNF) therapies (infliximab, adalimumab, and golimumab), anti-IL-12/23 agents (ustekinumab), and anti-integrin agents (vedolizumab) [15–19]. Tofacitinib, a small-molecule drug that inhibits the Janus kinase (JAK) family of proteins, is used to treat UC.

The European Crohn’s and Colitis Organisation (ECCO) guidelines indicate that similar efficacy and safety in luminal CD was observed in all currently available anti-TNF therapies. All anti-TNF agents were more effective than placebo in maintaining clinical remission, while infliximab was found to be more effective than adalimumab and certolizumab during the induction phase [20]. Vedolizumab can be considered as an alternative option for treating CD patients who have failed anti-TNF or immunosuppressant therapy, or who have moderate disease activity [20]. Ustekinumab has also shown some efficacy for inducing clinical response and clinical remission of active CD [20].

For acute severe UC, the ECCO guidelines suggest intravenous (IV) ciclosporin, tacrolimus, or infliximab as an alternative to patients with serious adverse events or fail to respond to IV corticosteroids [20]. For patients with UC who do not respond to or fail anti-TNF therapy, the British Society of Gastroenterology (BSG) guidelines recommend switching to alternative therapeutic choices such as vedolizumab and tofacitinib for induction and maintenance [21].

Infliximab has improved health outcomes for patients with IBD through its invasive delivery method, IV infusion while other anti-TNF therapies and biologics for IBD: adalimumab, ustekinumab, golimumab, and vedolizumab can all be administrated subcutaneously [15–19]. Although IV treatment may be more invasive than SC treatment, IV treatment imposes a financial burden, through resource use (equipment, outpatient bed space) and healthcare professional (HCP) time to prepare or administer infusions [22]. A study in Italy investigated the inpatient costs associated with a single infusion of infliximab in patients with IBD [23]. The mean total cost excluding drug cost was €250.86 for a standard 2-h infusion and €133.54 for a 1-h infusion. Another source in UK, the technology appraisal by the National Institute of Health and Care Excellence published an estimation of IV administration cost as £298 (around €348) [23, 24]. The largest driver of costs was time spent by nurses and specialists administering treatment and monitoring the patient after the infusion, accounting for almost 90% of all costs [23].

Route of delivery is recognized as an important factor associated with healthcare resource use and patient preference. Many patients have expressed a preference for subcutaneous (SC) administration over IV [25]. In a systematic review of patient preferences, four of six studies concluded that patients preferred SC rather than IV drug administration (range, 44–91% of respondents) [25]. The most frequent reasons for preferring SC treatment were ease of administration, less discomfort, and reduced time required for therapy [25].

The SC form of infliximab, infliximab SC, received market authorization from the European Medicines Agency (EMA) for rheumatoid arthritis in 2019 and IBD in 2020. Given that it is already prescribed in most E5 countries as a treatment for IBD, it is useful to quantify the potential savings associated with its use. A UK budget impact analysis of infliximab SC for rheumatoid arthritis noted significant cost savings [26]. Adding SC infliximab to the E5 market for IBD might similarly reduce associated healthcare costs such as HCP time and consumables for administration, while also optimizing work productivity for patients by reducing time spent on clinic visits [27]. We predicted the potential savings of IBD treatment by extending the market beyond the UK, to also include also the other four major countries of Europe (France, Germany, Italy, and Spain). We analysed the budget impact of introducing infliximab SC to the market in E5 countries where only IV infliximab was available. We propose two models, “world with infliximab SC” and “world without infliximab SC,” enabling HCPs and patients to assess the cost savings of using the SC formulation.

## Methods

### Model structure

A prevalence-based budget impact model (BIM) was developed to investigate the financial impact of infliximab SC with currently available biologics for IBD (including adalimumab, golimumab, infliximab, tofacitinib, vedolizumab, and ustekinumab) in the E5 market, taking the payer’s perspective.

The budget impact was measured using IQVIA MIDAS market share data. In the model, eligible patients for treatment and patient share of treatments were applied to calculate the magnitude of the impact. The two scenarios were then compared to perform the base-case analysis:

- “World without”—current treatment environment, without infliximab SC
- “World with”—infliximab SC is added to the existing treatment environment.

The net budget impact of adopting infliximab SC into the E5 market was the difference in the total cost between the “world without” and “world with” scenarios.

In each scenario, the number of patients was multiplied by the cost of each comparator and weighted by its relative frequency of use (an actual or estimated patient share) to yield total costs of each biologic treatment. The total costs were then compared between the two scenarios, generating an estimate of budget impact. In the base-case analysis, only drug acquisition costs were used; in the scenario analysis, administration costs were also included, to estimate the financial impact of infliximab SC from a healthcare payer’s perspective. Additional patients were calculated each year by dividing annual budget impact by the per-customer drug acquisition costs each year. The model calculated the budget impact of infliximab SC in a time horizon of 1–5 years. All analyses were performed using Microsoft Excel® 2016.

### Model assumptions

#### Dosing information and treatment

The dosage indicated in the Summary of Product Characteristics (SmPC) was used to treat adult patients with IBD for all treatments considered (Supplementary Table 1). All patients were assumed to have received an IV loading phase at treatment initiation (Year 1 only). For subsequent years, patients followed a maintenance treatment schedule. This meant that a single cohort was followed up for 5 consecutive years with their treatments. The model assumes that patient share in each year reflects drug persistence and switching patients; therefore, further adjustments were not made.

Although efficacy and safety are not equivalent among the treatments, no differences were assumed because the main goal of the study was to examine the budget savings of introducing infliximab, which has a long history of prescription and established similar/better efficacy and safety compared with other approved treatments in the market [28, 29]. Additionally, the safety and efficacy of IV versus SC infliximab were comparable in phase I and III trials [30]. Therefore, the model assumes market uptake of infliximab SC is realized without any medical concern.

#### Number of patients eligible for biologic treatments

Eligible patients for treatment were calculated using the population data and prevalence rate of CD and UC for each country, as well as patients eligible to receive biologics (18.20% for CD; 11.44% for UC). The patient eligibility rate was calculated based on the assumption that patients with CD and UC were eligible if they had moderately or severely active CD or UC with an inadequate response to conventional therapy (including corticosteroids, 6-mercaptopurine, or azathioprine) who were intolerant to or had medical contraindications for these therapies. Details used to calculate the eligibility rate for CD and UC are provided in Supplementary Table 2.

#### Patient share for pharmacological treatment

Market volume share data were extracted from IQVIA MIDAS, an analytics platform that has biologic sales data, to calculate patient shares of infliximab SC and its comparators in 2020 [31] (Supplementary Tables 3–12). The model captured market share from the following molecules: infliximab, adalimumab, and golimumab; and where available for each indication, tofacitinib, vedolizumab, and ustekinumab. In the model, infliximab SC was assumed to take over the market share from mostly infliximab IV products. Percentages in the “world without” scenario were based on sales data for the second quarter of 2020 and sustained throughout the 5-year period. The “world with” scenario assumed that infliximab SC takes 20% from all infliximab shares in Year 1, increasing 10% annually up to 50% in Year 5. In addition, it was assumed that infliximab SC will take 1% from adalimumab in Year 1, increasing 1% yearly up to 5% in Year 5. The 3% share of golimumab will be taken by infliximab SC, increasing 3% up to 15% in Year 5. For ustekinumab and vedolizumab, infliximab SC will take 2% share in Year 1, increasing 3% yearly up to 11% in Year 5. Lastly, no market share uptake for tofacitinib was assumed as it is delivered by the oral route. As a result of our assumptions, the share of infliximab SC ranged from 4.35% in Year 1 to 13.73% in Year 5.

The core elements we considered for the market share assumptions were the price of the drug in the market and the possibility of exchangeability between drugs in clinical practice. We assumed that infliximab SC will take over most shares of infliximab IV and small shares of ustekinumab and vedolizumab, as recommended in the ECCO guidelines for IBD [20, 32].

### Data sources

#### Population data

Population data, prevalence, and estimated number of patients eligible for biologics across the E5 countries were used to specify the target population for infliximab SC. Population data and prevalence rates for UC and CD were collected from the GlobalData [10, 33] [Supplementary Tables 13, 14].The patient eligibility rate was calculated under the assumption that patients with CD and UC were eligible if they had moderately or severely active CD or UC with an inadequate response to conventional therapy (including corticosteroids, 6-mercaptopurine, or azathioprine) and if they were intolerant of or had medical contraindications to these therapies. Direct costs, including drug acquisition and drug administration costs, were considered in the model. Moreover, the drug cost of infliximab SC was assumed to be equal to that of infliximab IV.

#### Cost data: drug acquisition

Drug acquisition costs of all the comparators were calculated using relevant drug cost data from local databases. The prices of available drugs and the pack prices were provided by the British National Formulary (BNF) for the UK [34]. For other countries, manufacturers’ drug list prices were extracted from the LAUER-TAXE database for Germany, Agenzia Italiana del Farmaco for Italy, French health insurance system database for France, and the Bot-PLUS database developed by the General Pharmaceutical Council for Spain [35–38]. A 3.5% discount rate to future costs was also applied, to reflect the costs and benefits accruing over the 5-year time horizon [39].

Total drug acquisition costs were calculated as follows. Average costs per unit had been calculated by multiplying mg per unit by the price per mg, then dose per year was multiplied to the average cost per unit. The prices for induction versus maintenance years are different, to reflect higher dose requirements in the first year of treatment. Infliximab IV treatment requires weight-based dosing, complicating calculation: patients are generally given a 5 mg/kg dose, so the number of vials required will vary. Since it is difficult to estimate all individual patients’ weight, a mean weight of patients with IBD in each E5 country was applied to calculate the vials required per patient. The mean weight of patients with IBD in the UK was 83.52 kg, 84.87 kg in Germany, 77.51 kg in France, 80.60 kg in Italy, and 76.99 kg in Spain [40]. Vial sharing was not considered in the analysis.

#### Cost data: administration

With regards to the E5 countries, payers will benefit from substituting IV administration with SC. In the UK for example, provider resources such as staff time, consumables, and overhead costs falls on the central payer for IV administration by offering set tariff. However, in the case of SC, 90–95% of drugs are administered by homecare services, which are funded by pharmaceutical companies, so that the NHS and providers do not bear the costs of SC administration. Administration costs for Germany were those reported by Einheitlicher Bewertungsmaßstab (EBM) published by the National Association of Statutory Health Insurance Physicians in Germany (KBV). In France, the T2A tariff list and the administration cost data were sourced from the Technical Agency for Information on Hospitalisation (Agence technique de l’information sur l’hospitalisation; ATIH). Hospitals charge €335 and private clinics charge €195 for IV administration, while SC does not incur any administration cost. In Italy, the central payer will ultimately benefit from reducing IV administration cases and switching to SC products. Hospital administrators will use fewer resources, consumables, chair use and overhead costs, but because these costs are reimbursed by central payer, the saving will be observed in the national payer level. In Spain, there were no official administration cost data that could be accessed for this study. Instead, costs were pulled out from a study conducted by José Andrés Román Ivorra, et al.

Administration costs were incorporated as a single cost input for each administration route. This cost input will account for the cost of all resources needed for administering a treatment. The costs of the actual administration include staff, equipment, and concomitant medication costs as well as proximal costs such as those associated with physician and clinic visits, training for self-administration, follow-up visits, and laboratory tests. The model assumes that there are no cost differences among the products with the same administration route but takes account of country-specific cost variations. All costs are inflated to 2021, and the unit costs were €365.07 in the UK, €294.80 in Germany, €320.24 in France, €381.66 in Italy, and €171.70 in Spain for IV administration when there is no cost associated with oral and SC administration [24, 41–43]. No administration costs for SC and oral treatments were applied.

## Results

### Base case: drug acquisition cost

The base-case model examines the budget impact of utilizing CTP-13 SC in the E5 countries for a 5-year time horizon. Tables 1 and 2 show the impact of introducing infliximab SC to CD and UC patients, respectively.

**Table 1:** Total net budget impact and potential additional patients by using infliximab SC for CD across the E5 countries: Base-case analysis

**Country**	**Year**	**Year 1**	**Year 2**	**Year 3**	**Year 4**	**Year 5**
UK	Budget impact (€)	-6,754,249	-3,233,133	-4,119,520	-4,939,899	-4,703,480
	Potential additional patients	643	307	406	504	497
Germany	Budget impact (€)	-21,742,039	-1,956,889	-2,293,718	-2,604,572	-1,984,305
	Potential additional patients	1,213	163	198	233	184
France	Budget impact (€)	-4,406,791	-2,383,420	-3,431,396	-4,403,093	-5,407,311
	Potential additional patients	839	451	673	895	1,139
Italy	Budget impact (€)	-6,297,818	-5,122,660	-6,735,146	-8,228,483	-8,524,351
	Potential additional patients	769	636	866	1,097	1,178
Spain	Budget impact (€)	-1,270,901	1,758,179	2,338,570	2,876,179	2,945,458
	Potential additional patients	106	-145	-200	-254	-270

**Table 2:** Total net budget impact and potential additional patients by using infliximab SC for UC across the E5 countries: Base-case model

**Country**	**Year**	**Year 1**	**Year 2**	**Year 3**	**Year 4**	**Year 5**
UK	Budget impact (€)	-7,671,259	-3,308,025	-4,262,503	-5,146,116	-5,038,627
	Potential additional patients	795	314	420	525	533
Germany	Budget impact (€)	-11,137,496	-3,964,392	-4,952,376	-5,866,345	-5,366,250
	Potential additional patients	621	220	285	350	331
France	Budget impact (€)	-4,469,157	-2,437,878	-3,503,293	-4,491,144	-5,500,340
	Potential additional patients	851	461	687	913	1,159
Italy	Budget impact (€)	-6,912,433	-6,090,567	-8,163,964	-10,084,761	-10,834,585
	Potential additional patients	844	756	1,050	1,344	1,497
Spain	Budget impact (€)	-848,667	985,414	1,309,142	1,609,003	1,643,488
	Potential additional patients	71	-81	-112	-142	-151

The model suggests that in the base case, introducing infliximab SC is cost-saving for the four of the E5 countries during the 5-year period in the treatment of both CD and UC. However, for Spain cost savings are incurred only during Year 1; from Year 2 onwards, overall spending increases if infliximab SC is introduced to patients. This trend is caused by the price of infliximab SC, assumption on the patient share over 5 years (reflecting estimated market uptake of infliximab SC), and posology difference. CT-13 SC in Spain has a higher list price than IV infliximab, based on the volume of active ingredients contained in each syringe or pen. The average annual cost of infliximab SC treatment is higher than adalimumab in Spain, which is widely used in the market for IBD patients. Moreover, the increasing market uptake of infliximab SC in Years 2–5 contributes towards an increase in overall budget.

### Scenario analysis: drug acquisition + administration costs

**Fig. 1:**
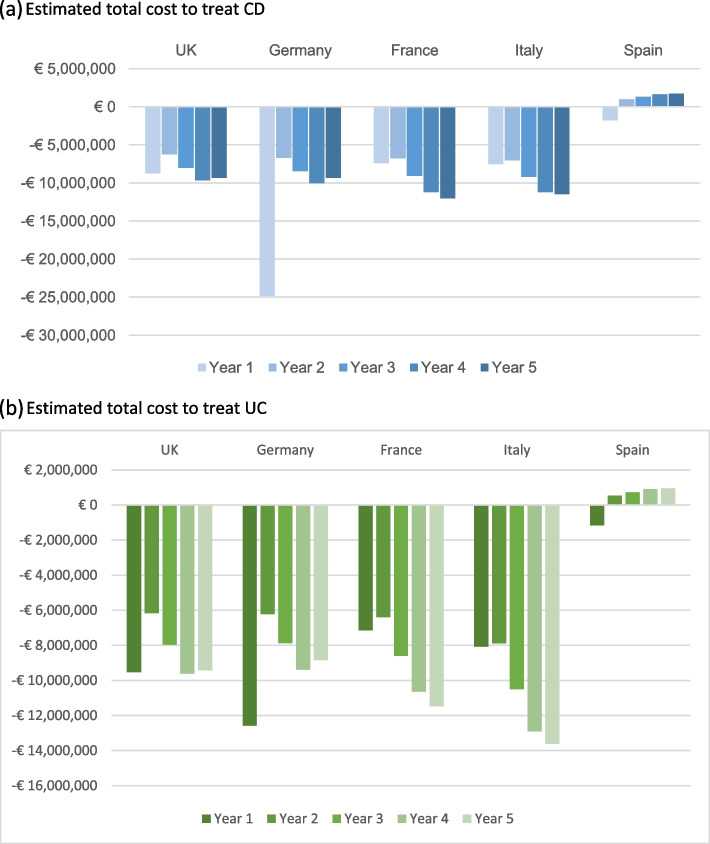
Summary of estimated total cost to treat CD and UC in all E5 countries. **a** Estimated total cost to treat CD. **b** Estimated total cost to treat UC

In the scenario analysis, administration costs were considered in addition to the base-case model. The potential financial benefits on total drug acquisition and administration cost expenses by utilizing infliximab SC are demonstrated in Tables 3 and 4. Similar to the base-case model, the scenario analysis suggests that using infliximab SC is cost-saving for all five European countries during the induction year. During the maintenance years, all countries except Spain were saving costs by utilizing infliximab SC. The impact on the Spanish healthcare budget improves through the scenario analysis compared with the base-case model. This indicates that although the drug acquisition cost might be higher than comparators, the total cost generated by switching from IV to SC infliximab might be lower than currently utilized products. Figure 1a and b provides a brief summary of the estimated total cost to treat CD and UC patients in E5 countries.

**Table 3:** Total net budget impact on using infliximab SC and potential additional patients for CD across the E5 countries: Scenario analysis

**Country**	**Year**	**Year 1**	**Year 2**	**Year 3**	**Year 4**	**Year 5**
UK	Budget impact (€)	-8,745,792	-6,257,880	-8,024,186	-9,659,184	-9,329,245
	Potential additional patients	832	595	790	986	986
Germany	Budget impact (€)	-24,815,419	-6,712,140	-8,444,598	-10,047,561	-9,348,132
	Potential additional patients	1,384	558	728	898	865
France	Budget impact (€)	-7,382,296	-6,751,710	-9,061,306	-11,200,960	-12,019,651
	Potential additional patients	1,406	1,278	1,777	2,277	2,532
Italy	Budget impact (€)	-7,532,192	-7,022,150	-9,192,415	-11,202,159	-11,468,029
	Potential additional patients	920	872	1,182	1,493	1,584
Spain	Budget impact (€)	-1,806,415	961,842	1,311,751	1,635,988	1,736,387
	Potential additional patients	151	-79	-112	-145	-159

**Table 4:** Total net budget impact on using infliximab SC and potential additional patients for UC across the E5 countries: Scenario analysis

**Country**	**Budget impact**	**Year 1**	**Year 2**	**Year 3**	**Year 4**	**Year 5**
UK	Budget impact (€)	-9,521,596	-6,165,237	-7,953,681	-9,609,381	-9,428,914
	Potential additional patients	987	586	783	980	997
Germany	Budget impact (€)	-12,580,068	-6,214,981	-7,863,495	-9,388,999	-8,851,437
	Potential additional patients	702	345	452	560	547
France	Budget impact (€)	-7,146,294	-6,390,907	-8,598,011	-10,642,789	-11,484,093
	Potential additional patients	1,361	1,210	1,686	2,163	2,419
Italy	Budget impact (€)	-8,066,236	-7,888,194	-10,489,458	-12,898,970	-13,620,404
	Potential additional patients	985	979	1,349	1,719	1,881
Spain	Budget impact (€)	-1,150,798	533,519	726,456	905,235	957,380
	Potential additional patients	96	-44	-62	-80	-88

## Discussion

This model estimates the financial impact of introducing SC infliximab for patients with IBD in E5 countries. Before infliximab SC was available, infliximab was only administered through the IV route, which required additional hospital resources such as facilities, equipment, and HCP time. Single-payer systems that previously provided coverage for drug and administration costs, such as in all E5 countries, are expected to incur savings from reduced administration costs following the introduction of SC infliximab.

The model concludes that the avoidance of IV administration costs will offset the higher drug acquisition cost of infliximab SC and lead to significant budget savings. As seen from the base-case model and scenario analysis, the inclusion of IV administration costs increased budget savings in France, Germany, Italy, and the UK. In Spain, reduced administration costs did not completely offset the increased drug acquisition cost but did reduce total spending on drug administration in both indications.

Some limitations are inherent in budget impact models. This BIM assumes that infliximab SC has the same efficacy and safety outcomes as IV infliximab treatments. This assumption is based on the results of a head-to-head trial that demonstrated comparable reductions in Disease Activity Score 28 (DAS 28) scores in patients receiving infliximab SC and IV infliximab from baseline to Week 30. Change in patient shares in the “world with infliximab SC” scenario is based on clinical assumption, given that retaining infliximab (arguably the most effective first-line biologic therapy in IBD) will impact the utilization of subsequent lines of biologic therapies. The model also assumes that infliximab SC will take around 2–11% share of the ustekinumab and vedolizumab markets. Typically, ustekinumab and vedolizumab are prescribed by clinicians when anti-TNF agents have either failed or been refused by patients. On this basis, it may be erroneous to assume that SC infliximab would take part of the market share for non–anti-TNF agents. However, given the limited treatment options in IBD, a further SC treatment option will increase the duration of anti-TNF treatment because patients will cycle through the anti-TNF class before moving on to a further line of biologics. Also, the model assumes less than absolute 1% change in market share changes in both vedolizumab and ustekinumab between “world with infliximab SC” and “world without infliximab SC,” representing a marginal effect on total budget.

The BIM simulation might not accurately reflect real-world clinical practice. First, this model does not account for dose escalation of infliximab, which often happens in the treatment of IBD. However, given that infliximab SC is not weight dependent and does not allow dose escalation, not accounting for IV dose escalation results in a conservative figure when calculating the net budget impact. Where more patients require IV dose escalation, more savings will be incurred, as they would be receiving fixed-dose SC formulations. Also, patients remain on the same treatment for the entire time horizon of the model, with no discontinuation or switching or mortality. The model assumes that 100% of patients will require induction treatment in Year 1 and all patients continue maintenance therapy from Years 2 to 5, thus changes in proportion of treatment-naïve patients or switching patients might produce different results. However, there is insufficient information in the literature to ascertain more precise numbers of patients requiring induction treatment or switching population. In addition, patient share assumption generated by IQVIA data already reflects the induction and switching populations. Also, discontinuation rates and mortality would be similar in the “world with” and “world without” infliximab scenarios, resulting in zero net benefit. We decided that this approach is valid because if this model were tailored to show budget savings at the hospital or regional level, setting 100% patients to start from induction treatment would be more suitable than identifying proportion of patients who require induction treatment.

Although this study explores cost-related impacts of utilizing infliximab SC and comparators, it does not reflect the true transaction cost of acquiring the products. Because market price and the magnitude of discounts are decided through a contract between the national health system and the manufacturers, the true market values are undisclosed. Thus, it was not considered in this model, and actual savings might differ from our findings.

For the calculation of IV administration costs, the model refers to cost studies conducted in each E5 country and applies the cost to all IV-type comparators regardless of product or indication [24, 41–43]. The estimated cost of infliximab administration in each country was uniformly applied across all IV products, because the IV market is mostly composed of infliximab and switching will mostly occur within existing IV infliximab patients. Yet, assumptions and references used to inform this calculation might not accurately reflect local market trends and could therefore under- or over-estimate the impact to the current healthcare system.

## Conclusion

The introduction of infliximab SC will achieve significant budget savings by alleviating the need for IV administration, thus reducing the costs of hospital resources and human labour.

## Supplementary Information

**Additional file 1. **

## Data Availability

All data analysed during this study are included in this published article and its resource files.

## Abbreviations

ATIH: Technical Agency for Information on Hospitalisation (Agence technique de l’information sur l’hospitalisation)
BIM: Budget impact model
BNF: British National Formulary
BSG: British Society of Gastroenterology
CD: Crohn's disease
DAS 28: Disease Activity Score 28
EBM: Uniform Evaluation Standard (Einheitlicher Bewertungsmaßstab)
ECCO: European Crohn's and Colitis Organization
EMA: European Medicines Agency
E5: Big-5 European countries, formerly called “EU-5” before the Brexit
HCP: Healthcare professional
IBD: Inflammatory bowel disease
IL: Interleukin
IV: Intrevenous
JAK: Janus kinase
KBV: National Association of Statutory Health Insurance Physicians in Germany (Kassenärztliche Bundesvereinigung)
SC: Subcutaneous
SmPC: Summary of product characteristics
TNF: Tumor necrosis factor
UC: Ulcerative colitis
UK: United Kingdom

## References

[1] de Mattos BR, Garcia MP, Nogueira JB, Paiatto LN, Albuquerque CG, Souza CL, Fernandes LG, Tamashiro WM, Simioni PU (2015). Inflammatory Bowel Disease: An Overview of Immune Mechanisms and Biological Treatments. Mediators Inflamm.

[2] Inflammatory bowel disease [https://www.nhs.uk/conditions/inflammatory-bowel-disease/.]

[3] Abraham C, Cho JH (2009). Inflammatory bowel disease. N Engl J Med.

[4] Kilby K, Mathias H, Boisvenue L, Heisler C, Jones JL (2019). Micronutrient absorption and related outcomes in people with inflammatory bowel disease: a review. Nutrients.

[5] Cosnes J, Gower-Rousseau C, Seksik P, Cortot A (2011). Epidemiology and natural history of inflammatory bowel diseases. Gastroenterology.

[6] Ananthakrishnan AN (2015). Epidemiology and risk factors for IBD. Nat Rev Gastroenterol Hepatol.

[7] Burisch J, Jess T, Martinato M, Lakatos PL (2013). The burden of inflammatory bowel disease in Europe. J Crohns Colitis.

[8] Molodecky NA, Soon IS, Rabi DM, Ghali WA, Ferris M, Chernoff G, Benchimol EI, Panaccione R, Ghosh S, Barkema HW (2012). Increasing incidence and prevalence of the inflammatory bowel diseases with time, based on systematic review. Gastroenterology.

[9] Ng SC, Shi HY, Hamidi N, Underwood FE, Tang W, Benchimol EI, Panaccione R, Ghosh S, Wu JCY, Chan FKL (2017). Worldwide incidence and prevalence of inflammatory bowel disease in the 21st century: a systematic review of population-based studies. Lancet.

[10] GBD Results Tool. In. Global Burden of Disease Data Resources. 2017. https://gbd2017.healthdata.org/gbd-search/. Accessed 4 Mar 2021.

[11] Chen QQ, Yan L, Wan J (2014). Select a suitable treatment strategy for Crohn's disease: step-up or top-down. EXCLI J.

[12] Leitner GC, Vogelsang H (2016). Pharmacological- and non-pharmacological therapeutic approaches in inflammatory bowel disease in adults. World J Gastrointest Pharmacol Ther.

[13] Garud S, Peppercorn MA (2009). Ulcerative colitis: current treatment strategies and future prospects. Therap Adv Gastroenterol.

[14] Paramsothy S, Rosenstein AK, Mehandru S, Colombel JF (2018). The current state of the art for biological therapies and new small molecules in inflammatory bowel disease. Mucosal Immunol.

[15] European Medicines Agency (2009). STELARA 130 mg concentrate for solution for infusion.

[16] European Medicines Agency (2009). Simponi 45 mg/0.45 mL solution for injection in pre-filled pen.

[17] European Medicines Agency (2009). Humira 20 mg solution for injection in pre-filled syringe.

[18] European Medicines Agency (2014). Entyvio 300 mg powder for concentrate for solution for infusion.

[19] European Medicines Agency (2009). Remicade 100 mg powder for concentrate for solution for infusion.

[20] Gomollon F, Dignass A, Annese V, Tilg H, Van Assche G, Lindsay JO, Peyrin-Biroulet L, Cullen GJ, Daperno M, Kucharzik T (2017). 3rd European Evidence-based Consensus on the Diagnosis and Management of Crohn's Disease 2016: Part 1: Diagnosis and Medical Management. J Crohns Colitis.

[21] Lamb CA, Kennedy NA, Raine T, Hendy PA, Smith PJ, Limdi JK, Hayee BH, Lomer MCE, Parkes GC, Selinger C (2019). British Society of Gastroenterology consensus guidelines on the management of inflammatory bowel disease in adults. Gut.

[22] Afzali A, Ogden K, Friedman ML, Chao J, Wang A (2017). Costs of providing infusion therapy for patients with inflammatory bowel disease in a hospital-based infusion center setting. J Med Econ.

[23] Mazzuoli S, Tricarico D, Demma F, Furneri G, Guglielmi FW (2016). Accelerated infliximab infusion: safety, factors predicting adverse events, patients' satisfaction and cost analysis. A Cohort Study in IBD Patients. PLoS One.

[24] Archer R, Tappenden P, Ren S, Martyn-St James M, Harvey R, Basarir H, Stevens J, Carroll C, Cantrell A, Lobo A (2016). Infliximab, adalimumab and golimumab for treating moderately to severely active ulcerative colitis after the failure of conventional therapy (including a review of TA140 and TA262): clinical effectiveness systematic review and economic model. Health Technol Assess.

[25] Stoner KL, Harder H, Fallowfield LJ, Jenkins VA (2015). Intravenous versus Subcutaneous Drug Administration. Which Do Patients Prefer? A Systematic Review. Patient.

[26] Byun HG, Jang M, Yoo HK, Potter J, Kwon TS (2021). Budget Impact Analysis of the Introduction of Subcutaneous Infliximab (infliximab SC) for the Treatment of Rheumatoid Arthritis in the United Kingdom. Appl Health Econ Health Policy.

[27] Tetteh EK, Morris S (2014). Evaluating the administration costs of biologic drugs: development of a cost algorithm. Health Econ Rev.

[28] Singh S, Fumery M, Sandborn WJ, Murad MH (2018). Systematic review and network meta-analysis: first- and second-line biologic therapies for moderate-severe Crohn's disease. Aliment Pharmacol Ther.

[29] Singh S, Murad MH, Fumery M, Dulai PS, Sandborn WJ (2020). First- and Second-Line Pharmacotherapies for Patients With Moderate to Severely Active Ulcerative Colitis: An Updated Network Meta-Analysis. Clin Gastroenterol Hepatol.

[30] A Phase I/III Study to Evaluate Efficacy, PK and Safety Between infliximab SC and infliximab IV in Patients With Active RA [https://clinicaltrials.gov/ct2/show/results/NCT03147248]

[31] MIDAS sales data. In. Edited by IQVIA; 2020.

[32] Harbord M, Eliakim R, Bettenworth D, Karmiris K, Katsanos K, Kopylov U, Kucharzik T, Molnár T, Raine T, Sebastian S (2017). Third European Evidence-based Consensus on Diagnosis and Management of Ulcerative Colitis. Part 2: Current Management. J Crohn's Colitis.

[33] United Kingdom population mid-year estimate [https://www.ons.gov.uk/peoplepopulationandcommunity/populationandmigration/populationestimates]

[34] Medicinal forms. In.: British National Formulary. https://bnf.nice.org.uk/medicinal-forms/infliximab.html. Accessed 12 Aug 2021.

[35] LAUER-TAXE®: reliable pharmaceutical information for all drugs and contracts registered in Germany. In.: Lauer-Taxe. https://portal.cgmlauer.cgm.com/LF/Seiten/Verwaltung/Kundencenter/1.aspx . Accessed 12 Aug 2021.

[36] BotPLUS Web. In.: BotPLUS. https://botplusweb.farmaceuticos.com/. Accessed 12 August 2021.

[37] Elenchi farmaci di classe A e H. In.: Agenzia Italiana del Farmaco (AIFA). https://www.aifa.gov.it/en/liste-farmaci-a-h. Accessed 12 Aug 2021.

[38] French health insurance system database. In.: L'assurance maladie. http://www.codage.ext.cnamts.fr/codif/bdm_it/index.php?p_site=AMELI. Accessed 12 Aug 2021.

[39] Sullivan SD, Mauskopf JA, Augustovski F, Jaime Caro J, Lee KM, Minchin M, Orlewska E, Penna P, Rodriguez Barrios JM, Shau WY (2014). Budget impact analysis-principles of good practice: report of the ISPOR 2012 Budget Impact Analysis Good Practice II Task Force. Value Health.

[40] WHO GloalHealthObservatory data [https://www.who.int/data/gho]

[41] Soini EJ, Leussu M, Hallinen T (2013). Administration costs of intravenous biologic drugs for rheumatoid arthritis. Springerplus.

[42] Principi M, Labarile N, Bianchi FP, Contaldo A, Tafuri S, Ierardi E, Di Leo A. The cost of inflammatory bowel disease management matches with clinical course: a single outpatient centre analysis. Int J Environ Res Public Health. 2020;17(12):4549. https://pubmed.ncbi.nlm.nih.gov/32599816/. 10.3390/ijerph17124549 PMC7344991 32599816

[43] Román Ivorra JA, Ivorra J, Monte-Boquet E, Canal C, Oyagüez I, Gómez-Barrera M. Cost analysis of biologic drugs in rheumatoid arthritis first line treatment after methotrexate failure according to patients’ body weight. Reumatol Clín (English Edition). 2016;12(3):123–9. 10.1016/j.reuma.2015.07.008 26362842

