# Supplementary material for: Budget impact analysis of the subcutaneous infliximab (CT-P13 SC) for treating inflammatory bowel disease in the Big-5 European (E5) countries
Source: BMC Health Serv Res. 2022 Nov 4;22:1319. doi: 10.1186/s12913-022-08683-y (PMC9636776; doi:10.1186/s12913-022-08683-y)
Supplement: Supplementary file 1 — Additional file 1. [file 12913_2022_8683_MOESM1_ESM.docx]

**Title:** Budget impact analysis of the subcutaneous infliximab (CT-P13 SC) for treating inflammatory bowel disease in the Big-5 European (E5) countries

**Journal**: *European Journal of Health Economics*

**Author names**: Hyun Kyeong Yoo, Han Geul Byun, Flavio Caprioli, Mathurin Fumery, Laurent Peyrin-Biroulet, Subramanian Sreedhar, James Potter, Minyoung Jang

**Funding:** The research was supported by Celltrion Healthcare.

**Conflict of interest:** HKY, HGB, JP and MYJ report that they have a financial interest in Celltrion Healthcare, a company that may be affected by the research reported in the enclosed paper. All authors are employed by Celltrion Healthcare.

**Corresponding author**: Minyoung Jang

Affiliation: Celltrion Healthcare, 19, Academy-ro 51, Yeonsu-gu, Incheon, South Korea

E-mail: MinYoung.Jang@celltrionhc.com

Supplementary Table 1 Dosing information from the SmPCs for adalimumab, golimumab, infliximab, tofacitinib, vedolizumab, and ustekinumab

| **INN** | **Indication** | **Recommended dose** | **Mode** | **Dosing** |
| --- | --- | --- | --- | --- |
| Infliximab [1] | CD & UC | 5 mg/kg | IV | - Induction: 5 mg/kg given as an intravenous infusion followed by an additional 5 mg/kg infusion 2 weeks after the first infusion. - Maintenance: additional infusion of 5 mg/kg at 6 weeks after the initial dose, followed by infusions every 8 weeks. |
| Infliximab [2] | CD & UC | 120 mg | SC | - Initiated as maintenance therapy 4 weeks after the last administration of two intravenous infusions of infliximab 5 mg/kg given 2 weeks apart. The recommended dose for Remsima subcutaneous formulation is 120 mg once every 2 weeks. |
| Adalimumab [3] | CD | Week 0: 80 mg  Week 2: 40 mg  Maintenance dose: 40 mg | SC | - Induction: 80 mg at Week 0 followed by 40 mg at Week 2. - After induction treatment, the recommended dose is 40 mg every other week via subcutaneous injection. |
|  | UC | Week 0: 160 mg  Week 2: 80 mg  Maintenance: 40 mg | SC | - 160 mg at Week 0 (given as four 40 mg injections in 1 day or as two 40 mg injections per day for 2 consecutive days) and 80 mg at Week 2 (given as two 40 mg injections in 1 day). - After induction treatment, the recommended dose is 40 mg every other week via subcutaneous injection. |
| Golimumab [4] | UC | Patients <80 kg:  Week 0: 200 mg  Week 2: 100 mg  Week 6: 50 mg  Maintenance: 50 mg  Patients ≥80 kg:  Week 0: 200 mg  Week 2: 100 mg  Maintenance: 100 mg | SC | - Patients with body weight <80 kg: given as an initial dose of 200 mg, followed by 100 mg at Week 2. Patients who have an adequate response should receive 50 mg at Week 6 and every 4 weeks thereafter. Patients who have an inadequate response may benefit from continuing with 100 mg at Week 6 and every 4 weeks thereafter. - Patients with body weight ≥80 kg:  given as an initial dose of 200 mg, followed by 100 mg at Week 2, then 100 mg every 4 weeks. |
| Vedolizumab [5] | CD & UC | 300 mg | IV | - 300 mg administered by intravenous infusion at 0, 2 and 6 weeks and then every 8 weeks thereafter. |
|  |  |  | SC | - Maintenance treatment following at least two intravenous infusions - 108 mg administered by subcutaneous injection once every 2 weeks. The first subcutaneous dose should be administered in place of the next scheduled intravenous dose and every 2 weeks. |
| Ustekinumab [6] | CD & UC | Approximately 6 mg/kg | IV & SC | - Initiated with a single intravenous dose based on body weight ≤55 kg: 260 mg >55 to ≤85 kg: 390 mg >85 kg: 520 mg. |
|  |  |  |  | - The first subcutaneous dose should be given at Week 8 following the intravenous dose. - The first subcutaneous administration of 90 mg should take place at Week 8 after the intravenous dose. After this, dosing every 12 weeks is recommended. |
| Tofacitinib [7] | UC | Induction: 10 mg twice daily  Maintenance: 5 mg twice daily | oral | - Induction: 10 mg given orally twice daily for induction for 8 weeks. - Maintenance: 5 mg given orally twice daily. |

Supplementary Table 2 Figures used to calculate the number of patients with IBD eligible for bDMARDs in the E5 countries

| ***A. Number of eligible patients with Crohn’s disease*** | | |
| --- | --- | --- |
| Indication criterion | **Result** | **Note** |
| Moderately to severely active Crohn's disease | 23.01% | Cosnes 2012 (France): https://gut.bmj.com/content/gutjnl/61/8/1140.full.pdf  Source data: people with severe CD (321/600) with active disease (median 43%) |
| Adult patients who have had an inadequate response to conventional therapy including corticosteroids and 6-mercaptopurine (6-MP) or azathioprine (AZA), or who are intolerant to or have medical contraindications for such therapies | 52.00% | Assumed the same as in Crohn’s disease.  The Cochrane Library 2016. Azathioprine or 6-mercaptopurine for induction of remission in Crohn’s disease (Review): https://www.cochranelibrary.com/cdsr/doi/10.1002/14651858.CD000545.pub5/epdf/standard  It is stated that: “There was no statistically significant difference in clinical remission rates between azathioprine or 6-mercaptopurine and placebo. Forty-eight per cent (95/197) of patients receiving antimetabolites achieved remission compared to 37% (68/183) of placebo patients (5 studies, 380 patients; RR 1.23, 95% CI 0.97 to 1.55).” |
| **Subtotal** | 11.96% |  |
| ***Of all people with fistulizing, active CD*** | 35.00% | Gecse 2013 (US): https://www.ncbi.nlm.nih.gov/pmc/articles/PMC4040755/ |
| ***B. Number of eligible patients with ulcerative colitis*** | | |
| Indication criterion | **Result** | **Note** |
| Moderately to severely active ulcerative colitis | 22.00% | The facts about inflammatory bowel diseases: https://www.crohnscolitisfoundation.org/sites/default/files/2019-02/Updated%20IBD%20Factbook.pdf |
| Adult patients who have had an inadequate response to conventional therapy including corticosteroids and 6-mercaptopurine (6-MP) or azathioprine (AZA), or who are intolerant to or have medical contraindications for such therapies | 52.00% | Assumed the same as in Crohn’s disease.  The Cochrane Library 2016. Azathioprine or 6-mercaptopurine for induction of remission in Crohn’s disease (Review): https://www.cochranelibrary.com/cdsr/doi/10.1002/14651858.CD000545.pub5/epdf/standard  It is stated that: “There was no statistically significant difference in clinical remission rates between azathioprine or 6-mercaptopurine and placebo. Forty-eight per cent (95/197) of patients receiving antimetabolites achieved remission compared to 37% (68/183) of placebo patients (5 studies, 380 patients; RR 1.23, 95% CI 0.97 to 1.55).” |
| **Of all people with UC** | 11.44% |  |

Supplementary Table 3 Patient share data of CD in the UK

| **World without CT-P13 SC [8]** | **Product** | **Year 1** | **Year 2** | **Year 3** | **Year 4** | **Year 5** | **World with CT-P13 SC** | **Product** | **Year 1** | **Year 2** | **Year 3** | **Year 4** | **Year 5** |
| --- | --- | --- | --- | --- | --- | --- | --- | --- | --- | --- | --- | --- | --- |
|  | Amgevita | 22.35% | 22.35% | 22.35% | 22.35% | 22.35% |  | Amgevita | 22.12% | 21.90% | 21.68% | 21.45% | 21.23% |
|  | Hulio | 0.00% | 0.00% | 0.00% | 0.00% | 0.00% |  | Hulio | 0.00% | 0.00% | 0.00% | 0.00% | 0.00% |
|  | Humira | 13.60% | 13.60% | 13.60% | 13.60% | 13.60% |  | Humira | 13.46% | 13.32% | 13.19% | 13.05% | 12.92% |
|  | Hyrimoz | 5.67% | 5.67% | 5.67% | 5.67% | 5.67% |  | Hyrimoz | 5.62% | 5.56% | 5.50% | 5.45% | 5.39% |
|  | Imraldi | 19.42% | 19.42% | 19.42% | 19.42% | 19.42% |  | Imraldi | 19.22% | 19.03% | 18.83% | 18.64% | 18.45% |
|  | Idacio | 0.13% | 0.13% | 0.13% | 0.13% | 0.13% |  | Idacio | 0.13% | 0.13% | 0.13% | 0.13% | 0.13% |
|  | Flixabi | 1.69% | 1.69% | 1.69% | 1.69% | 1.69% |  | Flixabi | 1.36% | 1.19% | 1.02% | 0.85% | 0.85% |
|  | Inflectra | 8.45% | 8.45% | 8.45% | 8.45% | 8.45% |  | Inflectra | 6.76% | 5.92% | 5.07% | 4.23% | 4.23% |
|  | Remicade | 0.95% | 0.95% | 0.95% | 0.95% | 0.95% |  | Remicade | 0.76% | 0.66% | 0.57% | 0.47% | 0.47% |
|  | Remsima | 7.72% | 7.72% | 7.72% | 7.72% | 7.72% |  | Remsima | 6.17% | 5.40% | 4.63% | 3.86% | 3.86% |
|  | Zessly | 1.45% | 1.45% | 1.45% | 1.45% | 1.45% |  | Zessly | 1.16% | 1.01% | 0.87% | 0.72% | 0.72% |
|  | Stelara | 9.89% | 9.89% | 9.89% | 9.89% | 9.89% |  | Stelara | 9.69% | 9.39% | 9.19% | 9.00% | 8.80% |
|  | Entyvio IV | 8.58% | 8.58% | 8.58% | 8.58% | 8.58% |  | Entyvio IV | 8.40% | 8.15% | 7.98% | 7.80% | 7.63% |
|  | Entyvio SC | 0.11% | 0.11% | 0.11% | 0.11% | 0.11% |  | Entyvio SC | 0.11% | 0.11% | 0.10% | 0.10% | 0.10% |
|  | CT-P13 SC | 0.00% | 0.00% | 0.00% | 0.00% | 0.00% |  | CT-P13 SC | 5.03% | 8.23% | 11.24% | 14.25% | 15.23% |

Supplementary Table 4 Patient share data of UC in the UK

| **World without**  **CT-P13 SC [8]** | **Product** | **Year 1** | **Year 2** | **Year 3** | **Year 4** | **Year 5** | **World with**  **CT-P13 SC** | **Product** | **Year 1** | **Year 2** | **Year 3** | **Year 4** | **Year 5** |
| --- | --- | --- | --- | --- | --- | --- | --- | --- | --- | --- | --- | --- | --- |
|  | Amgevita | 21.44% | 21.44% | 21.44% | 21.44% | 21.44% |  | Amgevita | 21.22% | 21.01% | 20.79% | 20.58% | 20.36% |
|  | Hulio | 0.00% | 0.00% | 0.00% | 0.00% | 0.00% |  | Hulio | 0.00% | 0.00% | 0.00% | 0.00% | 0.00% |
|  | Humira | 13.04% | 13.04% | 13.04% | 13.04% | 13.04% |  | Humira | 12.91% | 12.78% | 12.65% | 12.52% | 12.39% |
|  | Hyrimoz | 5.44% | 5.44% | 5.44% | 5.44% | 5.44% |  | Hyrimoz | 5.39% | 5.33% | 5.28% | 5.22% | 5.17% |
|  | Imraldi | 18.63% | 18.63% | 18.63% | 18.63% | 18.63% |  | Imraldi | 18.44% | 18.25% | 18.07% | 17.88% | 17.70% |
|  | Idacio | 0.13% | 0.13% | 0.13% | 0.13% | 0.13% |  | Idacio | 0.13% | 0.13% | 0.13% | 0.12% | 0.12% |
|  | Simponi | 2.09% | 2.09% | 2.09% | 2.09% | 2.09% |  | Simponi | 2.03% | 1.97% | 1.90% | 1.84% | 1.78% |
|  | Flixabi | 1.37% | 1.37% | 1.37% | 1.37% | 1.37% |  | Flixabi | 1.10% | 0.96% | 0.82% | 0.69% | 0.69% |
|  | Inflectra | 6.85% | 6.85% | 6.85% | 6.85% | 6.85% |  | Inflectra | 5.48% | 4.80% | 4.11% | 3.43% | 3.43% |
|  | Remicade | 0.77% | 0.77% | 0.77% | 0.77% | 0.77% |  | Remicade | 0.61% | 0.54% | 0.46% | 0.38% | 0.38% |
|  | Remsima | 6.26% | 6.26% | 6.26% | 6.26% | 6.26% |  | Remsima | 5.00% | 4.38% | 3.75% | 3.13% | 3.13% |
|  | Zessly | 1.17% | 1.17% | 1.17% | 1.17% | 1.17% |  | Zessly | 0.94% | 0.82% | 0.70% | 0.59% | 0.59% |
|  | Xeljanz | 2.16% | 2.16% | 2.16% | 2.16% | 2.16% |  | Xeljanz | 2.16% | 2.16% | 2.16% | 2.16% | 2.16% |
|  | Stelara | 11.84% | 11.84% | 11.84% | 11.84% | 11.84% |  | Stelara | 11.60% | 11.25% | 11.01% | 10.77% | 10.54% |
|  | Entyvio IV | 8.69% | 8.69% | 8.69% | 8.69% | 8.69% |  | Entyvio IV | 8.52% | 8.26% | 8.08% | 7.91% | 7.74% |
|  | Entyvio SC | 0.11% | 0.11% | 0.11% | 0.11% | 0.11% |  | Entyvio SC | 0.11% | 0.11% | 0.11% | 0.10% | 0.10% |
|  | CT-P13 SC | 0.00% | 0.00% | 0.00% | 0.00% | 0.00% |  | CT-P13 SC | 4.35% | 7.26% | 9.96% | 12.67% | 13.73% |

**Supplementary Table 5** Patient share data of CD in Germany

| **World without**  **CT-P13 SC [8]** | **Product** | **Year 1** | **Year 2** | **Year 3** | **Year 4** | **Year 5** | **World with**  **CT-P 13 SC** | **Product** | **Year 1** | **Year 2** | **Year 3** | **Year 4** | **Year 5** |
| --- | --- | --- | --- | --- | --- | --- | --- | --- | --- | --- | --- | --- | --- |
|  | Amgevita | 10.28% | 10.28% | 10.28% | 10.28% | 10.28% |  | Amgevita | 10.17% | 10.07% | 9.97% | 9.87% | 9.76% |
|  | Hulio | 7.68% | 7.68% | 7.68% | 7.68% | 7.68% |  | Hulio | 7.60% | 7.52% | 7.44% | 7.37% | 7.29% |
|  | Humira | 20.09% | 20.09% | 20.09% | 20.09% | 20.09% |  | Humira | 19.89% | 19.68% | 19.48% | 19.28% | 19.08% |
|  | Hyrimoz | 7.77% | 7.77% | 7.77% | 7.77% | 7.77% |  | Hyrimoz | 7.69% | 7.61% | 7.53% | 7.46% | 7.38% |
|  | Imraldi | 0.86% | 0.86% | 0.86% | 0.86% | 0.86% |  | Imraldi | 0.85% | 0.84% | 0.84% | 0.83% | 0.82% |
|  | Idacio | 9.84% | 9.84% | 9.84% | 9.84% | 9.84% |  | Idacio | 9.74% | 9.64% | 9.54% | 9.44% | 9.35% |
|  | Flixabi | 2.38% | 2.38% | 2.38% | 2.38% | 2.38% |  | Flixabi | 1.90% | 1.67% | 1.43% | 1.19% | 1.19% |
|  | Inflectra | 3.47% | 3.47% | 3.47% | 3.47% | 3.47% |  | Inflectra | 2.78% | 2.43% | 2.08% | 1.73% | 1.73% |
|  | Remicade | 6.22% | 6.22% | 6.22% | 6.22% | 6.22% |  | Remicade | 4.98% | 4.36% | 3.73% | 3.11% | 3.11% |
|  | Remsima | 2.14% | 2.14% | 2.14% | 2.14% | 2.14% |  | Remsima | 1.71% | 1.50% | 1.28% | 1.07% | 1.07% |
|  | Zessly | 0.73% | 0.73% | 0.73% | 0.73% | 0.73% |  | Zessly | 0.58% | 0.51% | 0.44% | 0.36% | 0.36% |
|  | Stelara | 16.37% | 16.37% | 16.37% | 16.37% | 16.37% |  | Stelara | 16.04% | 15.55% | 15.22% | 14.89% | 14.57% |
|  | Entyvio IV | 10.64% | 10.64% | 10.64% | 10.64% | 10.64% |  | Entyvio IV | 10.42% | 10.10% | 9.89% | 9.68% | 9.47% |
|  | Entyvio SC | 1.56% | 1.56% | 1.56% | 1.56% | 1.56% |  | Entyvio SC | 1.52% | 1.48% | 1.45% | 1.42% | 1.38% |
|  | CT-P13 SC | 0.00% | 0.00% | 0.00% | 0.00% | 0.00% |  | CT-P13 SC | 4.12% | 7.04% | 9.67% | 12.30% | 13.44% |

**Supplementary Table 6** Patient share data of UC in Germany

| **World without**  **CT-P13 SC [8]** | **Product** | **Year 1** | **Year 2** | **Year 3** | **Year 4** | **Year 5** | **World with**  **CT-P13 SC** | **Product** | **Year 1** | **Year 2** | **Year 3** | **Year 4** | **Year 5** |
| --- | --- | --- | --- | --- | --- | --- | --- | --- | --- | --- | --- | --- | --- |
|  | Amgevita | 9.03% | 9.03% | 9.03% | 9.03% | 9.03% |  | Amgevita | 8.94% | 8.85% | 8.76% | 8.67% | 8.58% |
|  | Hulio | 6.74% | 6.74% | 6.74% | 6.74% | 6.74% |  | Hulio | 6.67% | 6.61% | 6.54% | 6.47% | 6.41% |
|  | Humira | 17.64% | 17.64% | 17.64% | 17.64% | 17.64% |  | Humira | 17.47% | 17.29% | 17.12% | 16.94% | 16.76% |
|  | Hyrimoz | 6.82% | 6.82% | 6.82% | 6.82% | 6.82% |  | Hyrimoz | 6.75% | 6.69% | 6.62% | 6.55% | 6.48% |
|  | Imraldi | 8.64% | 8.64% | 8.64% | 8.64% | 8.64% |  | Imraldi | 8.56% | 8.47% | 8.38% | 8.30% | 8.21% |
|  | Idacio | 0.76% | 0.76% | 0.76% | 0.76% | 0.76% |  | Idacio | 0.75% | 0.74% | 0.73% | 0.73% | 0.72% |
|  | Simponi | 3.62% | 3.62% | 3.62% | 3.62% | 3.62% |  | Simponi | 3.51% | 3.41% | 3.30% | 3.19% | 3.08% |
|  | Flixabi | 2.09% | 2.09% | 2.09% | 2.09% | 2.09% |  | Flixabi | 1.67% | 1.46% | 1.25% | 1.05% | 1.05% |
|  | Inflectra | 3.05% | 3.05% | 3.05% | 3.05% | 3.05% |  | Inflectra | 2.44% | 2.13% | 1.83% | 1.52% | 1.52% |
|  | Remicade | 5.47% | 5.47% | 5.47% | 5.47% | 5.47% |  | Remicade | 4.37% | 3.83% | 3.28% | 2.73% | 2.73% |
|  | Remsima | 1.88% | 1.88% | 1.88% | 1.88% | 1.88% |  | Remsima | 1.50% | 1.32% | 1.13% | 0.94% | 0.94% |
|  | Zessly | 0.64% | 0.64% | 0.64% | 0.64% | 0.64% |  | Zessly | 0.51% | 0.45% | 0.38% | 0.32% | 0.32% |
|  | Xeljanz | 5.92% | 5.92% | 5.92% | 5.92% | 5.92% |  | Xeljanz | 5.92% | 5.92% | 5.92% | 5.92% | 5.92% |
|  | Stelara | 16.99% | 16.99% | 16.99% | 16.99% | 16.99% |  | Stelara | 16.65% | 16.14% | 15.80% | 15.46% | 15.12% |
|  | Entyvio IV | 9.34% | 9.34% | 9.34% | 9.34% | 9.34% |  | Entyvio IV | 9.16% | 8.88% | 8.69% | 8.50% | 8.32% |
|  | Entyvio SC | 1.37% | 1.37% | 1.37% | 1.37% | 1.37% |  | Entyvio SC | 1.34% | 1.30% | 1.27% | 1.24% | 1.22% |
|  | CT-P13 SC | 0.00% | 0.00% | 0.00% | 0.00% | 0.00% |  | CT-P13 SC | 3.78% | 6.53% | 9.00% | 11.47% | 12.63% |

**Supplementary Table 7** Patient share data of CD in France

| **World without**  **CT-P13 SC [8]** | **Product** | **Year 1** | **Year 2** | **Year 3** | **Year 4** | **Year 5** | **World with**  **CT-P13 SC** | **Product** | **Year 1** | **Year 2** | **Year 3** | **Year 4** | **Year 5** |
| --- | --- | --- | --- | --- | --- | --- | --- | --- | --- | --- | --- | --- | --- |
|  | Amgevita | 7.25% | 7.25% | 7.25% | 7.25% | 7.25% |  | Amgevita | 7.18% | 7.11% | 7.04% | 6.96% | 6.89% |
|  | Hulio | 2.26% | 2.26% | 2.26% | 2.26% | 2.26% |  | Hulio | 2.24% | 2.22% | 2.20% | 2.17% | 2.15% |
|  | Humira | 37.46% | 37.46% | 37.46% | 37.46% | 37.46% |  | Humira | 37.08% | 36.71% | 36.33% | 35.96% | 35.58% |
|  | Hyrimoz | 0.88% | 0.88% | 0.88% | 0.88% | 0.88% |  | Hyrimoz | 0.87% | 0.86% | 0.85% | 0.85% | 0.84% |
|  | Imraldi | 2.62% | 2.62% | 2.62% | 2.62% | 2.62% |  | Imraldi | 2.59% | 2.56% | 2.54% | 2.51% | 2.49% |
|  | Idacio | 0.66% | 0.66% | 0.66% | 0.66% | 0.66% |  | Idacio | 0.65% | 0.64% | 0.64% | 0.63% | 0.62% |
|  | Flixabi | 2.65% | 2.65% | 2.65% | 2.65% | 2.65% |  | Flixabi | 2.12% | 1.86% | 1.59% | 1.33% | 1.33% |
|  | Inflectra | 14.23% | 14.23% | 14.23% | 14.23% | 14.23% |  | Inflectra | 11.39% | 9.96% | 8.54% | 7.12% | 7.12% |
|  | Remicade | 7.63% | 7.63% | 7.63% | 7.63% | 7.63% |  | Remicade | 6.10% | 5.34% | 4.58% | 3.81% | 3.81% |
|  | Remsima | 5.38% | 5.38% | 5.38% | 5.38% | 5.38% |  | Remsima | 4.30% | 3.76% | 3.23% | 2.69% | 2.69% |
|  | Stelara | 12.79% | 12.79% | 12.79% | 12.79% | 12.79% |  | Stelara | 12.54% | 12.15% | 11.90% | 11.64% | 11.39% |
|  | Entyvio | 6.19% | 6.19% | 6.19% | 6.19% | 6.19% |  | Entyvio | 6.07% | 5.88% | 5.76% | 5.63% | 5.51% |
|  | CT-P13 SC | 0.00% | 0.00% | 0.00% | 0.00% | 0.00% |  | CT-P13 SC | 6.87% | 10.94% | 14.82% | 18.70% | 19.59% |

**Supplementary Table 8** Patient share data of UC in France

| **World without**  **CT-P13 SC [8]** | **Product** | **Year 1** | **Year 2** | **Year 3** | **Year 4** | **Year 5** | **World with**  **CT-P13 SC** | **Product** | **Year 1** | **Year 2** | **Year 3** | **Year 4** | **Year 5** |
| --- | --- | --- | --- | --- | --- | --- | --- | --- | --- | --- | --- | --- | --- |
|  | Amgevita | 6.61% | 6.61% | 6.61% | 6.61% | 6.61% |  | Amgevita | 6.55% | 6.48% | 6.42% | 6.35% | 6.28% |
|  | Hulio | 2.06% | 2.06% | 2.06% | 2.06% | 2.06% |  | Hulio | 2.04% | 2.02% | 2.00% | 1.98% | 1.96% |
|  | Humira | 34.15% | 34.15% | 34.15% | 34.15% | 34.15% |  | Humira | 33.81% | 33.47% | 33.13% | 32.79% | 32.44% |
|  | Hyrimoz | 0.80% | 0.80% | 0.80% | 0.80% | 0.80% |  | Hyrimoz | 0.79% | 0.79% | 0.78% | 0.77% | 0.76% |
|  | Imraldi | 2.39% | 2.39% | 2.39% | 2.39% | 2.39% |  | Imraldi | 2.36% | 2.34% | 2.31% | 2.29% | 2.27% |
|  | Idacio | 0.60% | 0.60% | 0.60% | 0.60% | 0.60% |  | Idacio | 0.59% | 0.59% | 0.58% | 0.58% | 0.57% |
|  | Simponi | 4.42% | 4.42% | 4.42% | 4.42% | 4.42% |  | Simponi | 4.29% | 4.15% | 4.02% | 3.89% | 3.76% |
|  | Flixabi | 2.42% | 2.42% | 2.42% | 2.42% | 2.42% |  | Flixabi | 1.94% | 1.69% | 1.45% | 1.21% | 1.21% |
|  | Inflectra | 12.98% | 12.98% | 12.98% | 12.98% | 12.98% |  | Inflectra | 10.38% | 9.08% | 7.79% | 6.49% | 6.49% |
|  | Remicade | 6.95% | 6.95% | 6.95% | 6.95% | 6.95% |  | Remicade | 5.56% | 4.87% | 4.17% | 3.48% | 3.48% |
|  | Remsima | 4.90% | 4.90% | 4.90% | 4.90% | 4.90% |  | Remsima | 3.92% | 3.43% | 2.94% | 2.45% | 2.45% |
|  | Xeljanz | 2.29% | 2.29% | 2.29% | 2.29% | 2.29% |  | Xeljanz | 2.29% | 2.29% | 2.29% | 2.29% | 2.29% |
|  | Stelara | 13.78% | 13.78% | 13.78% | 13.78% | 13.78% |  | Stelara | 13.50% | 13.09% | 12.82% | 12.54% | 12.26% |
|  | Entyvio | 5.64% | 5.64% | 5.64% | 5.64% | 5.64% |  | Entyvio | 5.53% | 5.36% | 5.25% | 5.14% | 5.02% |
|  | CT-P13 SC | 0.00% | 0.00% | 0.00% | 0.00% | 0.00% |  | CT-P13 SC | 6.44% | 10.34% | 14.06% | 17.77% | 18.76% |

**Supplementary Table 9** Patient share data of CD in Italy

| **World without**  **CT-P13 SC [8]** | **Product** | **Year 1** | **Year 2** | **Year 3** | **Year 4** | **Year 5** | **World with**  **CT-P13 SC** | **Product** | **Year 1** | **Year 2** | **Year 3** | **Year 4** | **Year 5** |
| --- | --- | --- | --- | --- | --- | --- | --- | --- | --- | --- | --- | --- | --- |
|  | Amgevita | 16.96% | 16.96% | 16.96% | 16.96% | 16.96% |  | Amgevita | 16.79% | 16.62% | 16.45% | 16.28% | 16.12% |
|  | Humira | 23.94% | 23.94% | 23.94% | 23.94% | 23.94% |  | Humira | 23.71% | 23.47% | 23.23% | 22.99% | 22.75% |
|  | Hyrimoz | 3.79% | 3.79% | 3.79% | 3.79% | 3.79% |  | Hyrimoz | 3.75% | 3.71% | 3.67% | 3.64% | 3.60% |
|  | Imraldi | 13.18% | 13.18% | 13.18% | 13.18% | 13.18% |  | Imraldi | 13.04% | 12.91% | 12.78% | 12.65% | 12.52% |
|  | Idacio | 0.47% | 0.47% | 0.47% | 0.47% | 0.47% |  | Idacio | 0.46% | 0.46% | 0.45% | 0.45% | 0.44% |
|  | Flixabi | 7.09% | 7.09% | 7.09% | 7.09% | 7.09% |  | Flixabi | 5.67% | 4.96% | 4.25% | 3.54% | 3.54% |
|  | Inflectra | 3.18% | 3.18% | 3.18% | 3.18% | 3.18% |  | Inflectra | 2.55% | 2.23% | 1.91% | 1.59% | 1.59% |
|  | Remicade | 1.37% | 1.37% | 1.37% | 1.37% | 1.37% |  | Remicade | 1.09% | 0.96% | 0.82% | 0.68% | 0.68% |
|  | Remsima | 3.00% | 3.00% | 3.00% | 3.00% | 3.00% |  | Remsima | 2.40% | 2.10% | 1.80% | 1.50% | 1.50% |
|  | Zessly | 0.81% | 0.81% | 0.81% | 0.81% | 0.81% |  | Zessly | 0.64% | 0.56% | 0.48% | 0.40% | 0.40% |
|  | Stelara | 14.95% | 14.95% | 14.95% | 14.95% | 14.95% |  | Stelara | 14.65% | 14.20% | 13.90% | 13.60% | 13.31% |
|  | Entyvio | 11.27% | 11.27% | 11.27% | 11.27% | 11.27% |  | Entyvio | 11.05% | 10.71% | 10.48% | 10.26% | 10.03% |
|  | CT-P13 SC | 0.00% | 0.00% | 0.00% | 0.00% | 0.00% |  | CT-P13 SC | 4.20% | 7.11% | 9.76% | 12.41% | 13.52% |

**Supplementary Table 10** Patient share data of UC in Italy

| **World without**  **CT-P13 SC [8]** | **Product** | **Year 1** | **Year 2** | **Year 3** | **Year 4** | **Year 5** | **World with**  **CT-P13 SC** | **Product** | **Year 1** | **Year 2** | **Year 3** | **Year 4** | **Year 5** |
| --- | --- | --- | --- | --- | --- | --- | --- | --- | --- | --- | --- | --- | --- |
|  | Amgevita | 15.13% | 15.13% | 15.13% | 15.13% | 15.13% |  | Amgevita | 14.98% | 14.83% | 14.68% | 14.53% | 14.38% |
|  | Humira | 21.36% | 21.36% | 21.36% | 21.36% | 21.36% |  | Humira | 21.15% | 20.94% | 20.72% | 20.51% | 20.30% |
|  | Hyrimoz | 3.38% | 3.38% | 3.38% | 3.38% | 3.38% |  | Hyrimoz | 3.35% | 3.31% | 3.28% | 3.24% | 3.21% |
|  | Imraldi | 11.76% | 11.76% | 11.76% | 11.76% | 11.76% |  | Imraldi | 11.64% | 11.52% | 11.40% | 11.29% | 11.17% |
|  | Idacio | 0.42% | 0.42% | 0.42% | 0.42% | 0.42% |  | Idacio | 0.41% | 0.41% | 0.40% | 0.40% | 0.39% |
|  | Simponi | 5.48% | 5.48% | 5.48% | 5.48% | 5.48% |  | Simponi | 5.31% | 5.15% | 4.98% | 4.82% | 4.66% |
|  | Flixabi | 6.32% | 6.32% | 6.32% | 6.32% | 6.32% |  | Flixabi | 5.06% | 4.43% | 3.79% | 3.16% | 3.16% |
|  | Inflectra | 2.84% | 2.84% | 2.84% | 2.84% | 2.84% |  | Inflectra | 2.27% | 1.99% | 1.70% | 1.42% | 1.42% |
|  | Remicade | 1.22% | 1.22% | 1.22% | 1.22% | 1.22% |  | Remicade | 0.98% | 0.85% | 0.73% | 0.61% | 0.61% |
|  | Remsima | 2.67% | 2.67% | 2.67% | 2.67% | 2.67% |  | Remsima | 2.14% | 1.87% | 1.60% | 1.34% | 1.34% |
|  | Zessly | 0.72% | 0.72% | 0.72% | 0.72% | 0.72% |  | Zessly | 0.57% | 0.50% | 0.43% | 0.36% | 0.36% |
|  | Xeljanz | 2.88% | 2.88% | 2.88% | 2.88% | 2.88% |  | Xeljanz | 2.88% | 2.88% | 2.88% | 2.88% | 2.88% |
|  | Stelara | 15.76% | 15.76% | 15.76% | 15.76% | 15.76% |  | Stelara | 15.44% | 14.97% | 14.66% | 14.34% | 14.03% |
|  | Entyvio | 10.06% | 10.06% | 10.06% | 10.06% | 10.06% |  | Entyvio | 9.86% | 9.56% | 9.35% | 9.15% | 8.95% |
|  | CT-P13 SC | 0.00% | 0.00% | 0.00% | 0.00% | 0.00% |  | CT-P13 SC | 3.96% | 6.79% | 9.37% | 11.95% | 13.15% |

**Supplementary Table 11** Patient share data of CD in Spain

| **World without**  **CT-P13 SC [8]** | **Product** | **Year 1** | **Year 2** | **Year 3** | **Year 4** | **Year 5** | **World with**  **CT-P13 SC** | **Product** | **Year 1** | **Year 2** | **Year 3** | **Year 4** | **Year 5** |
| --- | --- | --- | --- | --- | --- | --- | --- | --- | --- | --- | --- | --- | --- |
|  | Amgevita | 3.25% | 3.25% | 3.25% | 3.25% | 3.25% |  | Amgevita | 3.22% | 3.18% | 3.15% | 3.12% | 3.09% |
|  | Hulio | 0.05% | 0.05% | 0.05% | 0.05% | 0.05% |  | Hulio | 0.05% | 0.05% | 0.05% | 0.05% | 0.05% |
|  | Humira | 32.03% | 32.03% | 32.03% | 32.03% | 32.03% |  | Humira | 31.71% | 31.39% | 31.07% | 30.74% | 30.42% |
|  | Hyrimoz | 12.92% | 12.92% | 12.92% | 12.92% | 12.92% |  | Hyrimoz | 12.79% | 12.66% | 12.53% | 12.40% | 12.27% |
|  | Imraldi | 8.84% | 8.84% | 8.84% | 8.84% | 8.84% |  | Imraldi | 8.75% | 8.66% | 8.57% | 8.48% | 8.39% |
|  | Idacio | 0.05% | 0.05% | 0.05% | 0.05% | 0.05% |  | Idacio | 0.05% | 0.05% | 0.05% | 0.05% | 0.04% |
|  | Flixabi | 0.64% | 0.64% | 0.64% | 0.64% | 0.64% |  | Flixabi | 0.51% | 0.45% | 0.39% | 0.32% | 0.32% |
|  | Inflectra | 5.77% | 5.77% | 5.77% | 5.77% | 5.77% |  | Inflectra | 4.62% | 4.04% | 3.46% | 2.88% | 2.88% |
|  | Remicade | 5.41% | 5.41% | 5.41% | 5.41% | 5.41% |  | Remicade | 4.33% | 3.79% | 3.25% | 2.71% | 2.71% |
|  | Remsima | 6.48% | 6.48% | 6.48% | 6.48% | 6.48% |  | Remsima | 5.18% | 4.53% | 3.89% | 3.24% | 3.24% |
|  | Zessly | 1.71% | 1.71% | 1.71% | 1.71% | 1.71% |  | Zessly | 1.37% | 1.20% | 1.03% | 0.86% | 0.86% |
|  | Stelara | 17.48% | 17.48% | 17.48% | 17.48% | 17.48% |  | Stelara | 17.13% | 16.60% | 16.25% | 15.90% | 15.55% |
|  | Entyvio | 5.38% | 5.38% | 5.38% | 5.38% | 5.38% |  | Entyvio | 5.28% | 5.12% | 5.01% | 4.90% | 4.79% |
|  | CT-P13 SC | 0.00% | 0.00% | 0.00% | 0.00% | 0.00% |  | CT-P13 SC | 5.03% | 8.29% | 11.32% | 14.35% | 15.38% |

**Supplementary Table 12** Patient share data of UC in Spain

| **World without**  **CT-P13 SC [8]** | **Product** | **Year 1** | **Year 2** | **Year 3** | **Year 4** | **Year 5** | **World with**  **CT-P13 SC** | **Product** | **Year 1** | **Year 2** | **Year 3** | **Year 4** | **Year 5** |
| --- | --- | --- | --- | --- | --- | --- | --- | --- | --- | --- | --- | --- | --- |
|  | Amgevita | 2.93% | 2.93% | 2.93% | 2.93% | 2.93% |  | Amgevita | 2.90% | 2.87% | 2.85% | 2.82% | 2.79% |
|  | Hulio | 0.05% | 0.05% | 0.05% | 0.05% | 0.05% |  | Hulio | 0.05% | 0.05% | 0.04% | 0.04% | 0.04% |
|  | Humira | 28.91% | 28.91% | 28.91% | 28.91% | 28.91% |  | Humira | 28.62% | 28.33% | 28.05% | 27.76% | 27.47% |
|  | Hyrimoz | 11.66% | 11.66% | 11.66% | 11.66% | 11.66% |  | Hyrimoz | 11.55% | 11.43% | 11.31% | 11.20% | 11.08% |
|  | Imraldi | 7.98% | 7.98% | 7.98% | 7.98% | 7.98% |  | Imraldi | 7.90% | 7.82% | 7.74% | 7.66% | 7.58% |
|  | Idacio | 0.04% | 0.04% | 0.04% | 0.04% | 0.04% |  | Idacio | 0.04% | 0.04% | 0.04% | 0.04% | 0.04% |
|  | Simponi | 3.58% | 3.58% | 3.58% | 3.58% | 3.58% |  | Simponi | 3.47% | 3.36% | 3.26% | 3.15% | 3.04% |
|  | Flixabi | 0.58% | 0.58% | 0.58% | 0.58% | 0.58% |  | Flixabi | 0.46% | 0.41% | 0.35% | 0.29% | 0.29% |
|  | Inflectra | 5.21% | 5.21% | 5.21% | 5.21% | 5.21% |  | Inflectra | 4.17% | 3.65% | 3.13% | 2.60% | 2.60% |
|  | Remicade | 4.89% | 4.89% | 4.89% | 4.89% | 4.89% |  | Remicade | 3.91% | 3.42% | 2.93% | 2.44% | 2.44% |
|  | Remsima | 5.85% | 5.85% | 5.85% | 5.85% | 5.85% |  | Remsima | 4.68% | 4.09% | 3.51% | 2.92% | 2.92% |
|  | Zessly | 1.54% | 1.54% | 1.54% | 1.54% | 1.54% |  | Zessly | 1.24% | 1.08% | 0.93% | 0.77% | 0.77% |
|  | Xeljanz | 3.28% | 3.28% | 3.28% | 3.28% | 3.28% |  | Xeljanz | 3.28% | 3.28% | 3.28% | 3.28% | 3.28% |
|  | Stelara | 18.64% | 18.64% | 18.64% | 18.64% | 18.64% |  | Stelara | 18.27% | 17.71% | 17.33% | 16.96% | 16.59% |
|  | Entyvio | 4.86% | 4.86% | 4.86% | 4.86% | 4.86% |  | Entyvio | 4.76% | 4.62% | 4.52% | 4.42% | 4.33% |
|  | CT-P13 SC | 0.00% | 0.00% | 0.00% | 0.00% | 0.00% |  | CT-P13 SC | 4.71% | 7.84% | 10.74% | 13.64% | 14.73% |

**Supplementary Table 13** Population data for CD

| **Country** | **Total population [9]** | **Prevalence**  **[9]** | **Eligible for treatment with biologics** | **Total number of patients eligible for treatment with biologics** |
| --- | --- | --- | --- | --- |
| UK | 65,436,510 | 0.17% | 18.20% | 20,246 |
| Germany | 80,313,272 | 0.35% | 18.20% | 51,160 |
| France | 67,611,479 | 0.19% | 18.20% | 23,380 |
| Italy | 62,334,799 | 0.16% | 18.20% | 18,152 |
| Spain | 49,683,254 | 0.13% | 18.20% | 11,755 |

**Supplementary Table 14** Population data for UC

| **Country** | **Total population**  **[9]** | **Prevalence**  **[9]** | **Eligible for treatment with biologics** | **Total number of patients eligible for treatment with biologics** |
| --- | --- | --- | --- | --- |
| UK | 65,436,510 | 0.31% | 11.44% | 23,206 |
| Germany | 80,313,272 | 0.30% | 11.44% | 27,564 |
| France | 67,611,479 | 0.30% | 11.44% | 23,204 |
| Italy | 62,334,799 | 0.27% | 11.44% | 19,254 |
| Spain | 49,683,254 | 0.13% | 11.44% | 7,389 |

Supplementary Table 15 Cost of drugs in the UK [10]

| **Brand** | **Molecule** | **Type** | **Formulation** | **Price per mg** **(€)** | **Price per unit (€)** |
| --- | --- | --- | --- | --- | --- |
| Amgevita | Adalimumab | Biosimilar | Pre-filled pen | 7.92 | 350.93 |
| Hulio | Adalimumab | Biosimilar | Pre-filled pen | 7.70 | 341.32 |
| Humira | Adalimumab | Reference | Pre-filled pen | 8.80 | 390.07 |
| Hyrimoz | Adalimumab | Biosimilar | Pre-filled pen | 8.08 | 357.89 |
| Imraldi | Adalimumab | Biosimilar | Pre-filled pen | 7.92 | 351.07 |
| Idacio | Adalimumab | Biosimilar | Pre-filled pen | 7.92 | 351.07 |
| Simponi | Golimumab | Reference | Pre-filled pen | 15.26 | 845.16 |
| Flixabi | Infliximab IV | Biosimilar | Powder for concentrate for solution for infusion vials | 3.77 | 417.61 |
| Inflectra | Infliximab IV | Biosimilar | Powder for concentrate for solution for infusion vials | 3.78 | 418.34 |
| Remicade | Infliximab IV | Reference | Powder for concentrate for solution for infusion vials | 4.20 | 464.82 |
| Remsima | Infliximab IV | Biosimilar | Powder for concentrate for solution for infusion vials | 3.78 | 418.34 |
| Zessly | Infliximab IV | Biosimilar | Powder for concentrate for solution for infusion vials | 3.78 | 418.34 |
| Xeljanz | Tofacitinib | Reference | Tablet | 2.46 | 13.65 |
| Stelara IV | Ustekinumab | Reference | Concentrate for solution for infusion vials | 16.52 | 2,378.28 |
| Stelara SC | Ustekinumab | Reference | Pre-filled syringe | 23.86 | 2,378.28 |
| Entyvio IV | Vedolizumab IV | Reference | Powder for concentrate for solution for infusion vials | 6.83 | 2,270.83 |
| Entyvio SC | Vedolizumab SC | Reference | Pre-filled disposable injection (POM) | 4.75 | 567.71 |
| CT-P13 SC | Infliximab SC | - | Pre-filled syringe | 3.15 | 418.34 |

Supplementary Table 16 Cost of drugs in Germany [11]

| **Brand** | **Molecule** | **Type** | **Formulation** | **Price per mg (€)** | **Price per unit (€)** |
| --- | --- | --- | --- | --- | --- |
| Amgevita | Adalimumab | Biosimilar | Pre-filled pen | 11.64 | 477.82 |
| Hulio | Adalimumab | Biosimilar | Pre-filled pen | 11.64 | 477.80 |
| Humira | Adalimumab | Reference | Pre-filled pen | 23.29 | 494.91 |
| Hyrimoz | Adalimumab | Biosimilar | Pre-filled pen | 11.64 | 477.81 |
| Imraldi | Adalimumab | Biosimilar | Pre-filled pen | 11.64 | 477.82 |
| Idacio | Adalimumab | Biosimilar | Pre-filled pen | 11.64 | 474.99 |
| Simponi | Golimumab | Reference | Pre-filled pen | 39.86 | 989.81 |
| Flixabi | Infliximab IV | Biosimilar | Powder for concentrate for solution for infusion vials | 6.57 | 699.08 |
| Inflectra | Infliximab IV | Biosimilar | Powder for concentrate for solution for infusion vials | 6.85 | 702.73 |
| Remicade | Infliximab IV | Reference | Powder for concentrate for solution for infusion vials | 6.90 | 708.30 |
| Remsima | Infliximab IV | Biosimilar | Powder for concentrate for solution for infusion vials | 6.85 | 702.73 |
| Zessly | Infliximab IV | Biosimilar | Powder for concentrate for solution for infusion vials | 6.92 | 702.73 |
| Xeljanz | Tofacitinib | Reference | Tablet | 3.62 | 17.64 |
| Stelara IV | Ustekinumab | Reference | Concentrate for solution for infusion vials | 39.43 | 5,284.43 |
| Stelara SC | Ustekinumab | Reference | Pre-filled syringe | 56.95 | 5,284.43 |
| Entyvio IV | Vedolizumab IV | Reference | Powder for concentrate for solution for infusion vials | 8.23 | 2,532.07 |
| Entyvio SC | Vedolizumab SC | Reference | Pre-filled syringe | 5.86 | 649.20 |
| CT-P13 SC | Infliximab SC | - | Pre-filled syringe | 5.82 | 716.21 |

Supplementary Table 17 Cost of drugs in France [12]

| **Brand** | **Molecule** | **Type** | **Formulation** | **Price per mg (€)** | **Price per unit (€)** |
| --- | --- | --- | --- | --- | --- |
| Amgevita | Adalimumab | Biosimilar | Pre-filled pen | 10.76 | 430.35 |
| Hulio | Adalimumab | Biosimilar | Pre-filled pen | 10.76 | 430.35 |
| Humira | Adalimumab | Reference | Pre-filled pen | 14.35 | 573.80 |
| Hyrimoz | Adalimumab | Biosimilar | Pre-filled pen | 10.76 | 430.35 |
| Imraldi | Adalimumab | Biosimilar | Pre-filled pen | 10.76 | 430.35 |
| Idacio | Adalimumab | Biosimilar | Pre-filled pen | 10.76 | 430.35 |
| Simponi | Golimumab | Reference | Pre-filled pen | 13.33 | 666.67 |
| Flixabi | Infliximab IV | Biosimilar | Powder for concentrate for solution for infusion vials | 2.03 | 203.37 |
| Inflectra | Infliximab IV | Biosimilar | Powder for concentrate for solution for infusion vials | 2.03 | 203.37 |
| Remicade | Infliximab IV | Reference | Powder for concentrate for solution for infusion vials | 2.03 | 203.37 |
| Remsima | Infliximab IV | Biosimilar | Powder for concentrate for solution for infusion vials | 2.03 | 203.37 |
| Zessly | Infliximab IV | Biosimilar | Powder for concentrate for solution for infusion vials | 2.03 | 203.37 |
| Xeljanz | Tofacitinib | Reference | Tablet | 2.19 | 10.96 |
| Stelara IV & Stelara SC | Ustekinumab | Reference | Concentrate for solution for infusion vials | 16.43 | 2,135.47 |
| Stelara SC | Ustekinumab | Reference | Pre-filled syringe | 23.73 | 2,135.47 |
| Entyvio IV | Vedolizumab IV | Reference | Powder for concentrate for solution for infusion vials | 4.46 | 1,337.88 |
| CT-P13 SC | Infliximab SC | - | Pre-filled syringe | 1.69 | 210.00 |

Supplementary Table 18 Cost of drugs in Italy [13]

| **Brand** | **Molecule** | **Type** | **Formulation** | **Price per mg (€)** | **Price per unit (€)** |
| --- | --- | --- | --- | --- | --- |
| Amgevita | Adalimumab | Biosimilar | Pre-filled pen | 9.64 | 385.75 |
| Humira | Adalimumab | Reference | Pre-filled pen | 12.05 | 482.19 |
| Hyrimoz | Adalimumab | Biosimilar | Pre-filled pen | 8.56 | 342.36 |
| Imraldi | Adalimumab | Biosimilar | Pre-filled pen | 8.56 | 342.36 |
| Idacio | Adalimumab | Biosimilar | Pre-filled pen | 8.56 | 342.36 |
| Simponi | Golimumab | Reference | Pre-filled pen | 20.88 | 1,044.19 |
| Flixabi | Infliximab IV | Biosimilar | Powder for concentrate for solution for infusion vials | 3.86 | 386.28 |
| Inflectra | Infliximab IV | Biosimilar | Powder for concentrate for solution for infusion vials | 3.86 | 386.28 |
| Remicade | Infliximab IV | Reference | Powder for concentrate for solution for infusion vials | 4.64 | 463.53 |
| Remsima | Infliximab IV | Biosimilar | Powder for concentrate for solution for infusion vials | 3.86 | 386.28 |
| Zessly | Infliximab IV | Biosimilar | Powder for concentrate for solution for infusion vials | 3.86 | 386.28 |
| Xeljanz | Tofacitinib | Reference | Tablet | 2.57 | 12.87 |
| Stelara IV & Stelara SC | Ustekinumab | Reference | Concentrate for solution for infusion vials | 21.87 | 2,842.88 |
| Stelara SC | Ustekinumab | Reference | Pre-filled syringe | 31.59 | 2,842.88 |
| Entyvio IV | Vedolizumab IV | Reference | Powder for concentrate for solution for infusion vials | 6.79 | 2,036.04 |
| CT-P13 SC | Infliximab SC | - | Pre-filled syringe | 3.22 | 320.20 |

Supplementary Table 19 Cost of drugs in Spain [14]

| **Brand** | **Molecule** | **Type** | **Formulation** | **Price per mg (€)** | **Price per unit (€)** |
| --- | --- | --- | --- | --- | --- |
| Amgevita | Adalimumab | Biosimilar | Pre-filled pen | 7.84 | 313.50 |
| Hulio | Adalimumab | Biosimilar | Pre-filled pen | 7.84 | 313.50 |
| Humira | Adalimumab | Reference | Pre-filled pen | 7.84 | 313.50 |
| Hyrimoz | Adalimumab | Biosimilar | Pre-filled pen | 7.84 | 313.50 |
| Imraldi | Adalimumab | Biosimilar | Pre-filled pen | 7.84 | 313.50 |
| Idacio | Adalimumab | Biosimilar | Pre-filled pen | 7.84 | 313.50 |
| Simponi | Golimumab | Reference | Pre-filled pen | 20.66 | 1,033.23 |
| Flixabi | Infliximab IV | Biosimilar | Powder for concentrate for solution for infusion vials | 4.02 | 402.21 |
| Inflectra | Infliximab IV | Biosimilar | Powder for concentrate for solution for infusion vials | 4.02 | 402.21 |
| Remicade | Infliximab IV | Reference | Powder for concentrate for solution for infusion vials | 4.02 | 402.21 |
| Remsima | Infliximab IV | Biosimilar | Powder for concentrate for solution for infusion vials | 4.02 | 402.21 |
| Zessly | Infliximab IV | Biosimilar | Powder for concentrate for solution for infusion vials | 4.02 | 402.21 |
| Xeljanz | Tofacitinib | Reference | Tablet | 2.72 | 13.61 |
| Stelara IV & Stelara SC | Ustekinumab | Reference | Concentrate for solution for infusion vials | 22.06 | 2,867.50 |
| Stelara SC | Ustekinumab | Reference | Pre-filled syringe | 31.86 | 2,867.50 |
| Entyvio IV | Vedolizumab IV | Reference | Powder for concentrate for solution for infusion vials | 10.69 | 3,206.05 |
| CT-P13 SC | Infliximab SC | - | Pre-filled syringe | 4.02 | 482.65 |

**References**

1. Agency EM. Remicade 100 mg powder for concentrate for solution for infusion.: European Medicines Agency; 2009.

2. Agency EM. Remsima 120 mg solution for injection in pre-filled syringe.

3. Agency EM. Humira 20 mg solution for injection in pre-filled syringe. European Medicines Agency; 2009.

4. Agency EM. Simponi 45 mg/0.45 mL solution for injection in pre-filled pen.: European Medicines Agency; 2009.

5. Agency EM. Entyvio 300 mg powder for concentrate for solution for infusion. European Medicines Agency; 2014.

6. Agency EM. STELARA 130 mg concentrate for solution for infusion. European Medicines Agency; 2009.

7. Agency EM. XELJANZ film-coated tablets.

8. MIDAS sales data. In: IQVIA, editor.; 2020.

9. Evaluation IfHMa. GBD 2019 data. Global Health Data Exchange; 2019.

10. Formulary BN. Medicinal forms. 2021 [cited; Available from: <https://www.nice.org.uk/medicinal-forms/>

11. Lauer-Taxe. LAUER-TAXE®: reliable pharmaceutical information for all drugs and contracts registered in Germany. 2020 [cited; Available from: <https://www.gazzettaufficiale.it/eli/gu/2017/08/09/185/sg/pdf>

12. maladie La. French health insurance system database. 2020.

13. (AIFA) AIdF. Elenchi farmaci di classe A e H. 2020.

14. BotPLUS. BotPLUS Web. 2020.
